# Motor Experience Reprograms Development of a Genetically-Altered Bilateral Corticospinal Motor Circuit

**DOI:** 10.1371/journal.pone.0163775

**Published:** 2016-09-27

**Authors:** Najet Serradj, John H. Martin

**Affiliations:** 1 Department of Physiology, Pharmacology and Neuroscience, City University of New York School of Medicine, New York, NY, United States of America; 2 Neuroscience Program, Graduate Center of the City University of New York, New York, NY, United States of America; Stanford University School of Medicine, UNITED STATES

## Abstract

Evidence suggests that motor experience plays a role in shaping development of the corticospinal system and voluntary motor control, which is a key motor function of the system. Here we used a mouse model with conditional forebrain deletion of the gene for EphA4 (Emx1-Cre:EphA4^tm2Kldr^), which regulates development of the laterality of corticospinal tract (CST). We combined study of Emx1-Cre:EphA4^tm2Kldr^ with unilateral forelimb constraint during development to expand our understanding of experience-dependent CST development from both basic and translational perspectives. This mouse develops dense ipsilateral CST projections, a bilateral motor cortex motor representation, and bilateral motor phenotypes. Together these phenotypes can be used as readouts of corticospinal system organization and function and the changes brought about by experience. The Emx1-Cre:EphA4^tm2Kldr^ mouse shares features with the common developmental disorder cerebral palsy: bilateral voluntary motor impairments and bilateral CST miswiring. Emx1-Cre:EphA4^tm2Kldr^ mice with typical motor experiences during development display the bilateral phenotype of “mirror” reaching, because of a strongly bilateral motor cortex motor representation and a bilateral CST. By contrast, Emx1-Cre:EphA4^tm2Kldr^ mice that experienced unilateral forelimb constraint from P1 to P30 and tested at maturity had a more contralateral motor cortex motor representation in each hemisphere; more lateralized CST projections; and substantially more lateralized/independent reaching movements. Changes in CST organization and function in this model can be explained by reduced synaptic competition of the CST from the side without developmental forelimb motor experiences. Using this model we show that unilateral constraint largely abrogated the effects of the genetic mutation on CST projections and thus demonstrates how robust and persistent experience-dependent development can be for the establishment of corticospinal system connections and voluntary control. Further, our findings inform the mechanisms of and strategies for developing behavioral therapies to treat bilateral movement impairments and CST miswiring in cerebral palsy.

## Introduction

Expression of skilled voluntary movements during development depends on the establishment of functional connections between the motor cortex and spinal motor circuits and, in particular, those of the corticospinal tract (CST). Development of this motor pathway reflects an interplay between genetics and neural activity. Genetic mechanisms initially guide formation of the CST and its connections, in part, through specification of neuronal subtypes and the regional expression of guidance molecules [1–3]. The activity of the corticospinal system helps steer postnatal development of the topography of spinal connections and the efficacy of the CST in producing motor responses [4, 5].

Closely related to activity-dependent development, the role of early limb motor experience in CST development is poorly understood. Experience-dependent development comprises feed-forward signaling of spinal motor circuits, somatic sensory feedback produced during movement, and possibly the particular association of the two. It is known that development of the corticospinal system is affected by the loss of motor experiences during the early postnatal period [6, 7]. And that preventing limb use late during postnatal CST developmental reduces CST axon branching and presynaptic sites, reduces axon density in the dorsal horn, and impairs forearm supination control [6, 7]. However, our knowledge is limited because there have been few studies and the influence of experience on many important aspects of CST organization and function have yet to be examined.

In this report we use unilateral limb use restriction in a mouse model (Emx1-Cre:EphA4^tm2Kldr^) with conditional elimination of EphA4 in the forebrain [8] to expand our understanding of experience-dependent CST development from both a basic and a translational perspective. We chose this mouse model for three reasons. First, this mouse has a bilateral CST due to the loss of the CST response to spinal midline axon repulsion induced by forward EphrinB3 signaling [9, 10]. The bilateral CST offers a unique advantage for the study of experience-dependent CST development because we can determine if experience affects the laterality of the CST, a characteristic that determines independent upper limb use in humans and forelimb use in rodents. Wild-type animals have too few ipsilateral CST projections to inform effectively the role of experience. Second, in this model bilateral, or “mirror,” reaching depends on the bilateral CST phenotype [8]. As this mirror movement is a behavioral outcome of the altered CST projections in this model, it is strongly predicted that if there is a change in the laterality of the CST brought about by altered motor experience, the expression of mirror reaching will concomitantly change. Mirror movements are a characteristic feature of cerebral palsy, a common developmental movement disorder [11, 12]. Importantly, people with the hemiplegic form of cerebral palsy, typically produced by unilateral perinatal stroke, frequently have a bilateral corticospinal system that helps explain the mirror movements [13]. This mutant mouse thus models a key CST circuit change and motor impairment in cerebral palsy. Third, apart from genetic models, the presence of a dominant bilateral CST is only associated with the concurrent loss of the CST from the other hemisphere, such as after a large unilateral perinatal motor cortex lesion. This genetic model thus affords the unique opportunity to examine potential adaptive and maladaptive contributions of the ipsilateral and contralateral components of the CST to impairments in motor skills.

We addressed the question of whether restricting forelimb use unilaterally during early development alters the laterality of the CST, its terminal and preterminal axon morphology, the forelimb motor representation, and forelimb motor skills in maturity. We show that unilateral limb constraint during development produced a more lateralized CST and motor cortex motor map, as well as more independent/lateralized voluntary motor functions of the corticospinal system. Changes in CST organization and function in this model can be explained by reduced synaptic competition of the CST from the side without motor experiences. Further, our findings inform the mechanisms of, and strategies for developing behavioral therapies to treat, movement impairments in cerebral palsy.

## Materials and Methods

### Animals

All procedures were approved by the Institutional Animal Care and Use Committees of City College of the City University of New York. Experiments were conducted on neonates and adult mice. Two animal groups were used: one based on mice carrying a conditional allele of EphA4 (Emx1-Cre;EphA4^tm2Kldr^, hereafter referred to as EphA4 conditional knockout) provided as a heterozygous line by Dr. Klas Kullander [8, 14, 15] (MGI id: 4398683), and one group of wild type (WT) mice purchased from Jackson laboratory. In each group, control unrestrained animals were compared to animals in which one forelimb was restrained between P1 and P30. Heterozygous and homozygous offspring were identified through tail DNA PCR protocols, similar to our previous study [8]. S1 File presents a summary of body weights for the restrained and unrestrained wild type and EphA4 conditional knockout mice. The surgeries conducted in adult mice (axon tracing, cortical electrophysiology) were performed under general anesthesia with a mixture of Ketamine/Xylazine (100mg/10mg/kg, i.p). The animals were placed in a stereotaxic frame (*Kopf Instruments*) and body temperature maintained at 37°C by a warming plate (*Physitemp*, *TCAT-2LV*, *Kopf Instruments*). For tracing studies, mice were administrated an analgesic (Rimadyl^®^, 5mg/kg, s.c).

### Forelimb constraint in neonate

**Fig 1:**
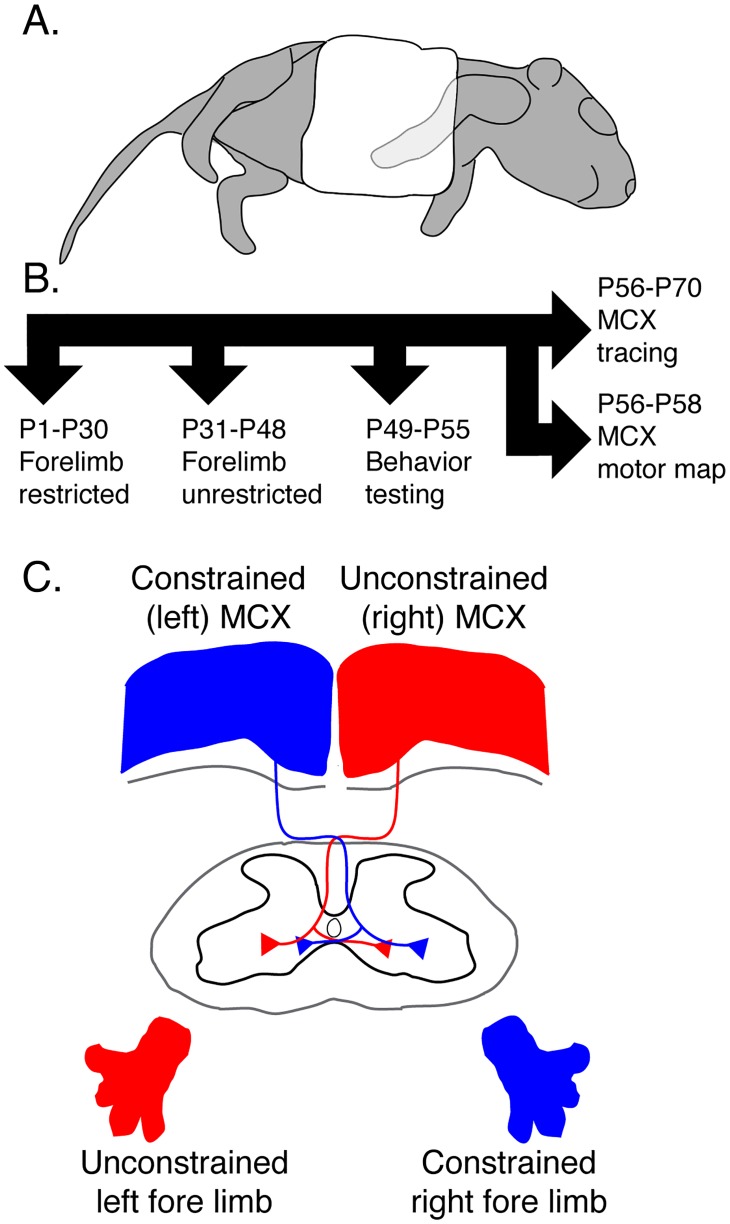
Unilateral forelimb restraint. A. Schematic showing a neonate mouse (P1) with unilateral constraint of the right forelimb against the chest using a surgical bandage tape. B. Experimental timeline. C. Experimental design. We traced separately the motor cortex (MCX) contralateral to the constrained right forelimb (termed constrained MCX; blue) and the MCX contralateral to the unconstrained left forelimb (termed unconstrained MCX; red).

Neonatal mice underwent unilateral forelimb constraint for a month beginning on P1; the constraint was removed on P30 (Fig 1A). The right forelimb was bandaged to the chest using surgical tape (3M Micropore). The neonates were returned to their mothers and checked twice daily; reapplication of the bandage was carried out as needed. As the mice grew we reinforced the surgical tape with tissue adhesive (3M^™^ Vetbond^™^). The constraint was removed at one month of age and animals recovered for 2 weeks or longer before their performance was tested on a battery of motor tasks. At the age of behavioral testing and euthanasia there were no differences in body weight between constrained wild type and EphA4 mice (see S1 File).

### Anatomical tracing

We used two different tracers for each hemisphere to identify the corticospinal tract axon labeling within the cervical enlargement gray matter. Biotinylated dextran amine (BDA 10.000MW, Molecular probes, 10% in 0.1M PB; 200nl/injection site) was microinjected (UMP3, World Precision Instruments) into one MCX and Dextran alexa fluor 488 (DAF 10.000MW, Molecular probes, 10% in 0.1_M_ PB; 200nl/injection site) in the contralateral MCX. S1 File lists tracers used for labeling CST axons in the two hemispheres and groups. For each tracer we made 3 injections, spaced by ~400μm (from bregma at AP = 0.4mm; ML = 1.2, 1.6, 2; DV = 0.8).

### Tissue preparation and staining

Two weeks later, mice were given an anesthetic overdose and perfused with heparinized saline followed by 4% paraformaldehyde in 0.1M PB. The brain and spinal cord were remove and post-fixed in the same fixative at room temperature for 2h then transferred to 20% sucrose in 0.1M PB overnight at 4°C. Frozen coronal sections through the cervical enlargement (C7/8) were cut at 40 μm for tracers histochemistry processing. To visualize BDA-labeled CST fibers, free-floating sections were incubated at room temperature for 1 h in 3% donkey serum with conjugated ExtrAvidin Cy3 (1:3000; Sigma). To visualize DAF 488-labeled fibers, sections were incubated with the primary antibody (1:400 rabbit anti-alexa Fluor 488; Molecular probes) overnight at 4°C. After washing with PBS 0.1_M_, sections were incubated in the secondary antibody at room temperature for 2h (1:500 donkey anti rabbit conjugated to FITC, Jackson ImmunoResearch). Sections were washed, mounted on gelatin-coated slides, air dried overnight, and cover slipped with Vectashield (Vector Laboratories).

### Anatomical data acquisition and analysis

#### Topographic analysis

For anterograde tracing, images at C7/8 were acquired on a Nikon inverted microscope under identical conditions of magnification, illumination and exposure to minimize variability. Images were converted to 8-bit black and white file format (S1 Fig; Photoshop; Adobe) and underwent a blinded digital analysis using a selective threshold adjustment to highlight only BDA/AF488-labeled axons. This process was the same as the one we have used previously ([16]; See Fig 1 in [17]. These images were then used to create topographical density maps (heat maps) to determine changes in ipsilateral and contralateral CST fiber projections. Axon distributions within the gray matter were analyzed in four transverse sections for each mouse. For construction of heat maps, digital analysis of individual sections were corrected for orientation and aligned with one another according to fiduciary marks (intersection between the gray matter above the central canal and the dorsal median septum). Photoshop TIFF files of individual sections of the spinal gray matter were skeletonized using ImageJ software so that each axon corresponded to a line one pixel thick. This important step is implemented in this this analysis so that the number and local density of pixels represent axon length, not axon width. Next, files were exported and analyzed using a custom program written in the Matlab (MathWorks), as previously described by [18]. S2 File presents the Matlab analysis script that was used. Briefly, Photoshop output files of individual sections of the spinal cord were divided into 80x80μm^2^ regions of interest (ROIs). For each ROI, we computed the mean density of BDA/AF488-labeled axons. A matrix of mean axon density was generated in Matlab that preserved the mediolateral and dorsoventral dimensions of the distribution of CST fibers labeled in the gray matter. Density is represented according to a color scale, from the lowest density (blue) to the highest (red). Regional distribution maps were generated for individual animals (e.g., see S2 Fig) and then averaged for all animals within each group.

Our anatomical data were corrected for variability in tracing efficacy between animals. Using the program Neurolucida (MBF, Bioscience) we counted the number of BDA/AF488-labeled axons into a 25x25μm^2^ ROI at the middle of the dorsal funiculus contralateral to the cortical injections. We divided individual section data (number of BDA/AF488-labeled axons of contralateral and ipsilateral gray matter) by the average of the estimated number of dorsal column axons per animal. The average of each animal was then divided by the average of the group to generate the correction factor for the animal’s data, as was done in our previous study [19]. The analysis script included a term for the user to enter the correction factor for each animal. In this way “heat maps” were corrected for tracer efficacy. Importantly, there were no differences in tracer efficacy for the two tracers (see S1 File). To analyze CST laterality, we quantified the total length of the CST labeled axons within the gray matter by measuring the total number of pixels on skeletonized images to single-pixel wide axons, using the program ImageJ. We divided the number of CST labeled axons measured in the ipsilateral by the total number of CST labeled axons in the gray matter to obtain a measure of system laterality.

#### Morphometric analysis of axon branching

We traced all labeled CS axon terminals within a square ROI (95μm x95μm) in the medial portion of the intermediate zone of the gray matter bilaterally, on four transverse sections through the cervical enlargement in each animal using Neurolucida (Microbrightfield, Inc). We chose this region because it consistently receives CST projections from each hemisphere, albeit denser ipsilateral projections in conditional knockout mice. A series of 30 optical slices, each 1 μm thick, comprising a z-stack image was collected through regions of interest and a projection image was constructed. Axons were reconstructed and the number of branch points and the length of each axon segment were measured. Limb disuse has been shown to reduce CST axon branching in the spinal cord [7, 20]. Further, in somatic sensory cortex axon branching decreases with reduced input (whisker trimming; peripheral nerve resection) and increases for spared inputs (e.g., [21]).

### Intracortical stimulation of motor cortex (M1)

For M1 stimulation experiments, we used similar parameters as we did for previous stimulation studies in mice [8, 14, 17, 22]. Briefly, we stimulated using tungsten microelectrodes (Microprobe, Inc.; 0.1 MΩ impedance; 81 μm shaft diameter, 1–2 μm tip diameter). Electrode penetrations were made perpendicular to the pial surface at the depths 0.8–0.9 mm. In all animals, the stimulated region was the same, between 0.0 to 2.4 mm lateral to bregma and up to 2 mm rostral to bregma. At each site, a 45ms train of 14 separate 200μs biphasic pulses was delivered at 330Hz from an isolated, constant current stimulator (model 2100, A-M Systems) at a rate of 0.5 Hz. The threshold was defined as the lowest current that produced a contralateral motor effect. A maximal current of 100 μA was used. If no response was evoked at or below 100 μA, the site was considered nonresponsive. For analysis, we computed the number of sites evoking motor responses. Sites were referenced to its stereotaxic coordinate. The probability of evoking a particular response at a particular site was computed for animals in each group.

### Training and motor behavioral testing

Motor performance during the behavioral tests were videotaped at 60 fields/s, using a Canon digital video camcorder (ZR 960, 41X zoom) at a shutter speed 1/500s with 280W illumination. For each animal, the video file was imported into a video-editing program (iMovie, Apple Macintosh computer) and viewed as single video frames at 30 Hz. Experimenters blinded to the group genotyping analyzed the recordings of testing sessions.

#### Exploratory reaching behavior

To assess reaching, animals were placed in a clear glass cylinder as we studied previously [8]. We videotaped and scored 30 reaching movements toward the cylinder wall.

#### Grasping test

We assessed the ability to grasp a thin horizontal steel bar (diameter: 2mm) for each paw separately by alternately constraining the right or left forepaw with surgical tape reinforced with tissue adhesive. To avoid incorrect positioning, the mouse was assisted until grasping firmly the bar. The test was performed on a single day with 15 trials and an inter-trial interval of 1 min. The latency to falling was scored for each trial.

#### Grid walking

We assessed each animal’s ability to coordinate forepaw placement during spontaneous locomotion on a wire grid floor (19x19 cm^2^, 0.8x0.8cm^2^ grid squares) placed above a glass surface, raised 70cm from the floor. Prior to video recording, each mouse was allowed to traverse the grid runway freely for 2mn. The mouse was filmed from below for 15min. A placement error was scored when the paw slipped through the grid opening. Thirty steps were scored for each forelimb for each recording session.

#### Locomotor behavior

Treadmill walking was chosen to gain insights into the CST/ MCX control during locomotion over obstacles placed on the treadmill belt (adaptive locomotion; [22, 23]). The mice were given 2 min to acclimate to the treadmill environment, while the treadmill remained stationary. During adaptive locomotion mice stepped over obstacles (1 cm height) equally mounted and spaced on the treadmill belt. Video recordings were analyzed for assessment of responses. We described stepping as an alternate movement between the right and the left limb, and hopping as bilateral limb movements when they lift and contact the treadmill belt. We scored a set of 30-cleared sequence over the obstacle.

### Statistical analysis

All statistical tests were carried out with GraphPad Prism software 5.0. Parametric tests were used when possible. Intergroup comparisons were tested either with a one way-ANOVA, with group as the independent factor and Bonferroni’s post- hoc analysis, or with Kruskal-Wallis test. Comparison between two groups was evaluated by either a *t*-test or the Mann-Whitney analysis. Intergroup comparisons were tested with a two-way ANOVA. All data are presented as the mean ± s.e.m. *P* values <0.05 were considered statistically significant. Supporting data files are available containing values used for plotting graphs in all figures (S3 File).

## Results

In this study, we addressed the question of whether restricting forelimb use unilaterally during early development, to reduce patterned motor system activity on the constrained side, alters development of the laterality of the CST, its terminal and preterminal axon morphology, and forelimb motor skills in maturity. This study was carried out in conditional EphA4 knockout mice (Emx1-Cre;EphA4^tm2Kldr^; [8] that have extensive ipsilateral CST projections and bilateral motor phenotypes, as well as wild-type mice. We prevented forelimb use unilaterally in neonatal EphA4 conditional knockout and wild-type mice between postnatal day P1 and P30 (Fig 1A and 1B). Two types of controls were used in this study: 1) EphA4 conditional knockout mice that received no constraint (typically termed EphA4 conditional knockout controls); and 2) wild-type mice that received no constraint. In mature mice subjected to unilateral forelimb constraint, we traced CST projections from the forelimb area of motor cortex (MCX) contralateral to the constrained forelimb (termed constrained MCX) and contralateral to the unconstrained forelimb (termed unconstrained MCX, Fig 1C). We determined changes in the MCX motor maps in each hemisphere due to unilateral limb constraint. We tested performance in a battery of motor tasks to assay the laterality and efficacy of motor skills. In wild-type and EphA4 conditional knockout mice that did not receive unilateral constraint only one hemisphere was traced. We focus initially on changes in the conditional knockout mice, with a description of changes in wild type mice at the end of the Results section.

### Early postnatal limb constraint abrogates the ipsilateral CST misprojections in conditional EphA4 knockout mice

**Fig 2:**
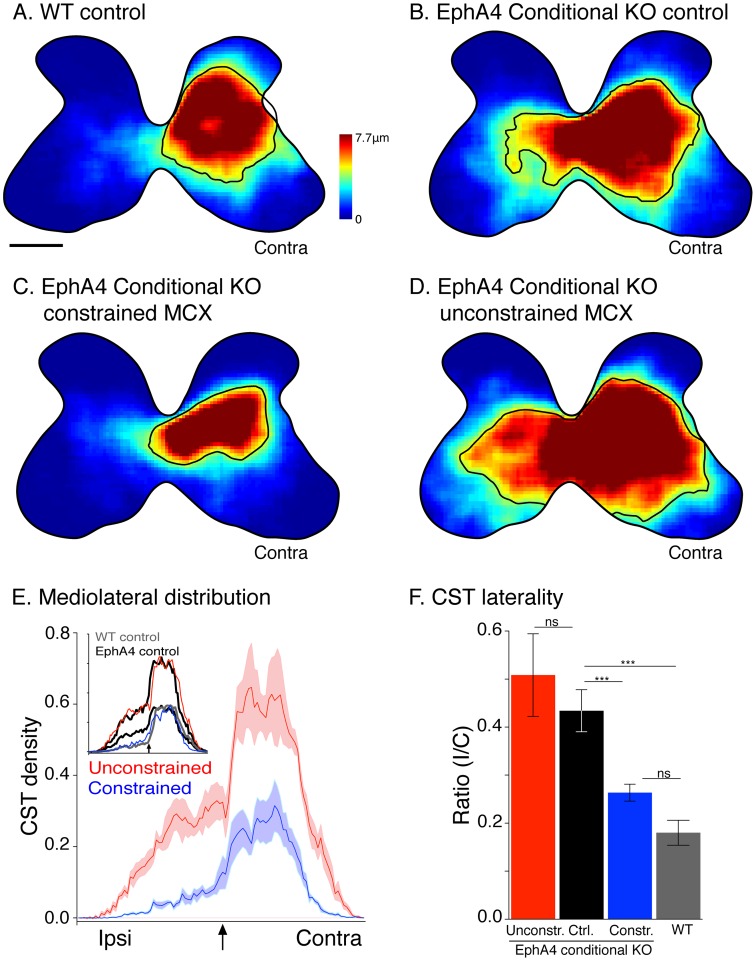
Topographic distribution of labeled CST axon terminals at C7/8. Averaged heat maps for mice (n = 5) in each group and condition (A-D). EphA4 conditional knockout controls (B) show extensive ipsilateral misprojections. Constraint of one forelimb caused a significant decrease in the density of CST projections from constrained MCX (C) shifting the distribution toward a pattern similar to WT group (A). CST projections from unconstrained MCX show a bilateral increase (D). Contours enclosing the region of highest density of labeling (the border between yellow and green on the heat maps) are shown for each condition. E. Mediolateral distributions of mean axon density from unconstrained MCX (red) and constrained MCX (blue) within gray matter. The y-axis plots the regional density of CST projections. Note the substantial ipsilateral projection from the unconstrained MCX that is as dense as the contralateral projection from the constrained MCX. Light shading plots ±SEM. The arrow indicates the midline. Inset compares unconstrained distribution (red line) with EphA4 conditional knockout control (black), scaled in amplitude to match that of the unconstrained distribution. Constrained distribution (blue line) with EphA4 conditional knockout control, scaled in amplitude to match that of the constrained distribution. The gray line is the distribution of WT controls. Control data replotted from [8]. F. Bar graphs plot the average laterality index (measured as ratio of ipsilateral gray matter labeling divided by contralateral labeling for MCX on each side). Data show a robust bilateral projection in EphA4 conditional knockout control mice with experience (black) due to the abundant ipsilateral CST misprojections (one-way ANOVA, *p*<0.0001, F_3, 71_ = 30.18; Bonferroni post-test: *p*<0.05). The unconstrained MCX reveals no significant changes (red), the constrained MCX shows a robust decrease in the aberrant ipsilateral terminations leading to a more contralateral organization (blue) similar to WT group (gray) (Bonferroni post-test: *p*>0.05). Constr. MCX.: Constrained motor cortex, unconstr. MCX: unconstrained motor cortex, Ctrl.: control. The color bar represents the axon length in micrometers within in each region of interest. Calibration (A) for heatmaps: 250 μm.

To evaluate whether motor experience altered the patterns of CST projections, we first constructed color-coded topographic “heat” maps of the average local density of CST labeling in the cervical enlargement (C7-C8; Fig 2A–2D). All heat maps are normalized for tracing efficacy in each animal and plotted with the same color scale. Whereas unconstrained wild type controls (Fig 2A) and unconstrained EphA4 conditional knockout control mice (2B) have similar contralateral CST projections, EphA4 unconstrained controls have extensive ipsilateral projections. This is consistent with the loss of the CST response to spinal midline axon repulsion signaling [9, 10]. The CST from the constrained MCX in EphA4 conditional knockout mice (serving the constrained right forelimb) had a reduced contralateral projection and the ipsilateral CST was nearly eliminated (Fig 2C). By contrast, CST projections from the unconstrained MCX (serving the unconstrained left forelimb) were increased bilaterally (Fig 2D). Montage images of labeling from one hemisphere of single representative animals are shown in S1 Fig These results were consistent across animals; tracing from each of the constrained hemispheres were strongly contralateral, whereas tracing from each unconstrained hemisphere expanded contralaterally and ipsilaterally (S2 Fig).

The mediolateral distribution of CST labeling (Fig 2E; red, projections from unconstrained MCX; blue, constrained MCX) in the conditional knockout mice reveals the enhanced projections bilaterally from the unconstrained MCX and near total loss of the ipsilateral projection from the constrained MCX. An important question is if the changes in CST projections—increases, as well as decreases, in ipsilateral and contralateral projections—were in proportion with one another or if some changes were disproportionately greater. To address this question we first compared the form of the medio-lateral CST gray matter axon distributions between groups, by scaling the heights of the EphA4 conditional knockout control distributions to reveal similarities in their shapes (Fig 2E, inset). The expanded bilateral termination field of the unconstrained cortex (inset; thin red line) has the same distribution as that of the EphA4 conditional knockout control with experience when the amplitude of the EphA4 control distribution is scaled up to match the peak of the EphA4 unconstrained contralateral distribution (ie., the black and red lines match). By contrast, when the EphA4 conditional knockout control distribution is now scaled down to match the peak contralateral value of the constrained side, we see that there are fewer ipsilateral projections than predicted by the scaled distribution. Importantly, the WT distribution (gray) matches the constrained contralateral distribution (i.e., gray and blue distributions match). We next compared changes in axon distributions due to limb constraint as a percent of the control CST projections in EphA4 conditional knockouts with experience. The percent increases in the contralateral (87±25.2%) and ipsilateral (106±24.9%) projections from the unconstrained MCX over control EphA4 values were not different (*p* = 0.66 paired *t-test*). In contrast, the percent reduction in the ipsilateral (68.6±7.1%) projection from the constrained cortex was significantly greater than the reduction in the contralateral (45.6±11.4) projection (*p* = 0.0094 paired *t-test*). This finding suggests that the contralateral and ipsilateral expansions of the unconstrained MCX/CST were in proportion with one another, but that the reduction in the ipsilateral CST from the constrained hemisphere was disproportionately greater than the reduction in contralateral CST from the constrained hemisphere.

Do these modifications in CST projections produced by unilateral constraint result in a CST that has a more contralateral anatomical organization? To answer this question we computed a laterality index for the CST from the constrained and unconstrained MCX (ipsilateral gray matter labeling divided by contralateral labeling for the MCX in each hemisphere; Fig 2F). Wild type mice have a small laterality index, owing to the paucity of ipsilateral CST projections (Fig 2F). EphA4 conditional knockout mice controls (black bar) have a significantly larger index relative to the wild type mice (one-way ANOVA, *p*<0.0001, F_3, 71_ = 30.18; Bonferroni post-test: *p*<0.05) due to the abundant ipsilateral CST misprojections. The CST from the constrained MCX showed a significant reduction in the laterality index compared with control EphA4 conditional knockout mice, and was not significantly different from the WT mice (Fig 2F; Bonferroni post-test: *p*>0.05). By contrast, the unconstrained MCX, despite the expanded CST projection, did not show a significant change in its laterality index (Bonferroni post-test: *p*>0.05). Our findings show a significantly more contralateral CST projection from the constrained MCX due to the preferential loss of ipsilateral projections. The CST from the unconstrained MCX did not become more bilateral, reflecting proportional increases in both ipsilateral and contralateral projections.

### Limb constraint changed CS Axon terminations morphology

**Fig 3:**
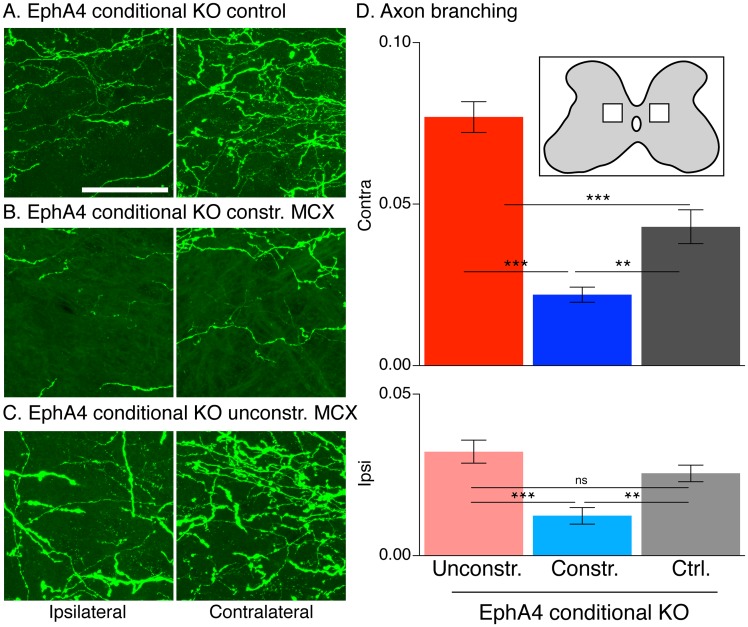
Forelimb constraint causes dual changes in CS axon terminals morphology. A-C confocal projection stacked image (30 optical slices). CST axon morphology in EphA4 conditional KO control (A), constrained MCX (B), and unconstrained MCX (C); ipsilateral (left) and contralateral (right) CST. D. Bar graphs plot CST axon branching within ROIs (inset; average of 4–5 mice/group, 4 sections/animal). Contralateral CST axon morphology on the constrained side (blue) of the spinal cord (right) revealed an overall significant difference between the groups (one-way ANOVA, *p*<0.0001, F_2,51_ = 37.70). Bonferroni posthoc testing revealed a significant 50% reduction in the mean number of contralateral CST axon branch points per μm originating from constrained MCX (blue) and controls (dark gray, *p<*0.05). Importantly, the contralateral projections of the unconstrained MCX (red) showed a significant 1.8 times increase in the mean axonal branch points relative to EphA4 control conditional knockout mice (dark gray). There was no difference in the ipsilateral branches for the unconstrained M1 (light red) relative to EphA4 control conditional knockout mice (light gray) (one-Way ANOVA, *p*<0.0001, F_2,51_ = 11.71; *p*>0.05, Bonferroni posthoc) but ipsilateral branching from the constrained MCX (light blue) was significantly less that either in controls (*p*<0.05, Bonferoni posthoc) or from the unconstrained side (*p*<0.05, Bonferoni posthoc). The inset shows the ROIs (95μm x95μm) we analyzed CS axon terminals morphology. Calibration: scale bar: 50μm.

We next determined if limb constraint also affected CST axon morphology in the spinal cord. We conducted a morphometric analysis of CST axons within a 95 μm X 95 μm ROI in the medial portion of the intermediate zone of the gray matter bilaterally (Fig 3D, inset). Thirty micrometer thick Z-stacks were collected, followed by reconstruction of individual axons, and identifying branch points. We chose this region because it consistently receives projections from each hemisphere in both the knockout (dense ipsilateral) and control (sparse ipsilateral). Representative photomicrographs within the ROI (A-C) show that, in relation to EphA4 conditional knockout controls, the CST from the constrained MCX lost axon branches whereas the CST from the unconstrained MCX gained axon branches. We quantified these changes by counting the number of CST axon branch points for each group. Contralateral CST axon morphology on the constrained side of the spinal cord (right) revealed an overall significant difference between the groups (Fig 3D; one-way ANOVA, *p*<0.0001, F_2,51_ = 37.70). Bonferroni posthoc testing revealed a significant reduction in the mean number of contralateral CST axon branch points per μm axon length from the constrained MCX and controls (*p<*0.05). Ipsilateral branching from the constrained MCX was significantly less that either in controls (*p*<0.05, Bonferoni posthoc) or from the unconstrained side (*p*<0.05, Bonferoni posthoc). By contrast, the contralateral projections of the unconstrained MCX showed a significant increase in the mean axonal branch points relative to EphA4 conditional knockout control mice (Fig 3D). Interestingly, there was no difference in the ipsilateral branches for the unconstrained MCX relative to EphA4 conditional knockout control mice (one-Way ANOVA, *p*<0.0001, F_2,51_ = 11.71; *p*>0.05, Bonferroni posthoc test). Our results show parallel changes in CST axon branching and topography for the constrained MCX. For the unconstrained MCX, the absence of increased ipsilateral branching may help to make this side somewhat more contralateral in its function.

### Limb constraint reduces motor cortex mirror sites in conditional EphA4 knockout mice

**Fig 4:**
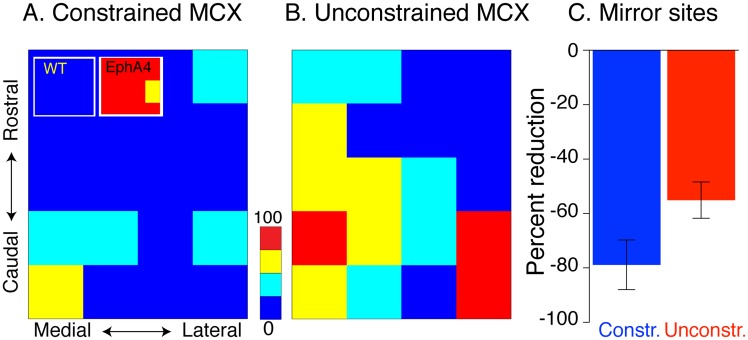
Forelimb constraint reduces mirror sites in EphA4 conditional knockout mice. A, B. Color maps plot the occurrence of evoked mirror movements at each MCX site. The percent of mirror sites is represented according to a color scale, from the lowest (blue) to the highest (red). The constrained MCX (A) shows a propensity of sites where mirror movements were not evoked (blue) and no sites were mirror movements were evoked most frequently (red). The unconstrained MCX (B) also showed a substantial reduction in mirror sites and a paucity of mirror movement sites. C. Bar graphs plot the average (n = 8 mice; 25 MCX sites within each of 16 hemispheres) of the percentage of sites from which the microstimulation evoked a mirror response. There was a 79% decrease in mirror sites from the constrained MCX (blue) and a 55% reduction in the unconstrained MCX (red) compared with EphA4 conditional knockout controls (*p* = 0.0004 Mann-Whitney test). The inset shows no mirror sites found in the WT at the threshold (all ‘‘blue” sites), whereas nearly all sites in EphA4 conditional knockout controls evoked mirror movements (mostly “red” sites); reanalyzed from data in [8].

Our findings show a robust reorganization of the CSTs from each hemisphere when one forelimb is constrained during postnatal development. The disproportionate reduction in aberrant ipsilateral termination density from the constrained MCX and loss of axon branching show that the CST from the constrained hemisphere shifts to a more contralateral organization. The increase in contralateral axon branching from the unconstrained MCX, without either a significant topographic laterality change or increased ipsilateral branching, suggest a shift to a more contralateral organization from that hemisphere as well. To determine whether these changes are physiologically important, we used intracortical microstimulation (ICMS) of the cortical forelimb motor representation. We determined if the MCX motor map in each hemisphere became more contralateral, similar to what we found for the topographic and morphological changes in CST projections. In each animal we stimulated 25 sites within the forelimb MCX area, and we distinguished those sites where the ipsilateral and contralateral evoked forelimb responses were at the same joint and were obtained at the same current threshold (termed ‘mirror movement’ sites) from sites in which there was only a contralateral forelimb movement at threshold. Ensemble motor maps (Fig 4A and 4B) plot the probability of evoking a mirror movement at each MCX site in relation to all evoked responses at that coordinate in all animals, according to a color scale. As we have previously shown for animals with normal limb experiences during development, wild type mice have no sites in which mirror movements are evoked (see inset Fig 4A; all “blue” sites; [8]) and EphA4 control conditional knockout mice have 80% of sites where mirror movements are evoked (inset Fig 4A; mostly “red” sites; [8]). Unilateral limb constraint significantly reduced aberrant mirror movements evoked by M1 stimulation in both hemispheres. The constrained MCX (Fig 4A) showed a majority of sites where mirror movements were not evoked (blue) and no sites were mirror movements were evoked most frequently (red). The unconstrained MCX (B) also showed a substantial reduction in mirror sites and a paucity of mirror movement sites, predicted on the basis of the strong increase in contralateral CST axon branching (Fig 3D). Both the constrained and unconstrained MCX showed remarkable reductions in mirror movement sites compared with EphA4 conditional knockout controls (Fig 4C; constrained: 79% reduction; unconstrained: 55% reduction; *p* = 0.0004 Mann-Whitney test). This robust reduction in the representation of mirror movements after unilateral limb constraint was not associated with a generalized change in the current threshold to evoke a contralateral movement from the constrained or unconstrained MCX compared with EphA4 conditional knockouts with limb experience (One way ANOVA, p = 0.59). Interestingly, there was an increase in the threshold for evoking ipsilateral movements from the constrained MCX (One way ANOVA, p = 0.003; Bonferoni post-test p<0.05). This result also stresses that the ipsilateral projections from the constrained MCX were particularly vulnerable to loss, whereas the contralateral projections were maintained within normal limits. Our findings demonstrate that unilateral constraint produced a robust shift from strongly bilateral MCX motor maps in each hemisphere to significantly more contralateral maps.

### Unilateral limb constraint reduces bilateral exploratory reaching movements in conditional EphA4 knockout mice

**Fig 5:**
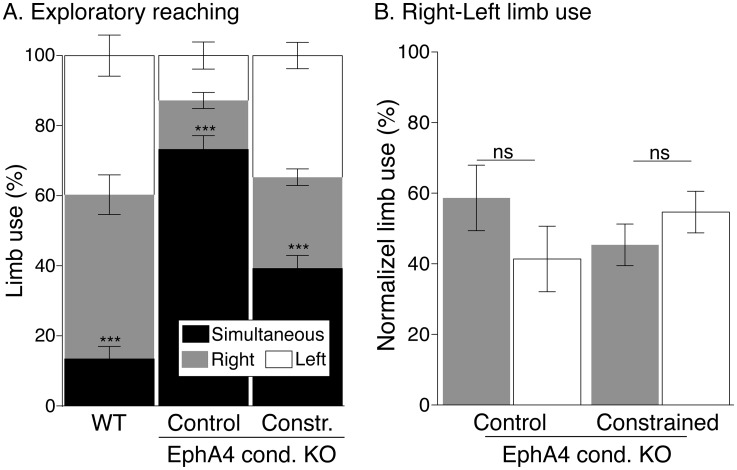
Forelimb constraint reduces bilateral reaching movements in EphA4 conditional knockout mice. A. Stacked bar graphs plot the average forelimb use (n = 10–13 mice/group) during exploratory reaching behavior. Wild type mice show a small incidence of simultaneous use of both forelimbs in this task (black, left bar) In contrast, EphA4 conditional knockout control mice use mirror reaching movements nearly 80% of the time (black, middle bar). Unilateral forelimb constraint caused approximately a 50% reduction in mirror movements while reaching the cylinder wall (black right bar). The overall difference between the groups was highly significant (one-Way ANOVA, *p*<0.0001, F_2.31_ = 57.70). Bonferroni post-hoc testing revealed a significant difference between EphA4 constrained and conditional knockout control mice (*p*<0.05), indicating significantly more independent forelimb use during reaching. However, there still was increased mirroring compared with wild type mice (Bonferroni posthoc: *p*<0.05,). B. Bar graphs plot the normalized mean value of independent forelimb use. We found no significant change in right-left forelimb use in the EphA4 constrained mice (Fig 5B) (*p* = 0.44 paired *t-test*) and EphA4 conditional knockout control mice (*p* = 0.37 paired *t-test*). Thus, the reduction in mirror reaching movements is due to improved independent limb use not a failure to use one limb.

The motor map in MCX is regarded to represent the capacity for voluntary motor skills [18, 24]. The strongly bilateral motor map in EphA4 control conditional knockout mice likely underlies the expression of mirror movements in voluntary control tasks [8]. We hypothesized that with a reduction in the number of sites evoking bilateral limb (mirror) movements, there would be a concomitant increase in independent forelimb use during voluntary control tasks. We tested the laterality of motor performance in mature mice (see Fig 1, timeline) by assessing forelimb exploratory reaching. This test allows the mouse to use either one or both forelimbs to reach the walls of a cylindrical testing chamber. Wild type mice show a small incidence of simultaneous use of both forelimbs in this task (black; 13.4±3.6) (Fig 5A). In contrast, EphA4 conditional knockout control mice use mirror reaching movements nearly 80% of the time [8]. Unilateral limb constraint caused approximately a 50% reduction in mirror movements while reaching the cylinder wall, (from 73.21±3.9% in EphA4 conditional knockout controls to 39.2±3.8% in EphA4 constrained mice). The overall difference between the groups was highly significant (one-Way ANOVA, *p*<0.0001, F_2.31_ = 57.70). Bonferroni post-hoc testing revealed a significant difference between EphA4 constrained and unconstrained control mice (*p*<0.05), indicating significantly more independent forelimb use during reaching. However, there still was increased mirroring compared with wild type mice (*p*<0.05, Bonferroni posthoc test). We wanted to ensure that the improvement in forelimb reaching movement was due to improved independent limb use and not due to favoring use of one limb at the expense of the other. For each mouse, we normalized the value of independent forelimb use to 100% and we computed the score for each limb. We found no significant change in right-left forelimb use in the EphA4 constrained mice (Fig 5B) (*p* = 0.44 paired *t-test*) and EphA4 conditional knockout control mice (*p* = 0.37 paired *t-test*). Thus, the reduction in mirror reaching movements is due to improved independent limb use not a failure to use one limb. This behavioral change can be explained by greater contralateral MCX motor maps in each hemisphere and a more effective contralateral projections from each MCX because of disproportionate loss of ipsilateral projections or gain of contralateral axon branching.

Another motor behavior that receives significant MCX/CST control is stepping over obstacles during treadmill locomotion (obstructed locomotion; Drew, 1991; Asante et al., 2010). We previously found that the conditional EphA4 knockout mice hop (i.e., simultaneous use of both forelimbs and/or hind limbs) over the obstacles, rather than use alternate stepping like WT mice (47.2±5.9% in EphA4 knockout and 1.5±0.7 in WT mice; [8]. Although we also expected greater independent limb use in this task (i.e., reduced hopping) after limb constraint, we were surprised that there was no significant change (S3 Fig). This difference between reaching and locomotion likely reflects differential spinal circuitry for the two behaviors, in which the performance in the obstructed locomotor task recruits spinal central pattern generator circuits that normally have robust bilateral connections whereas reaching does not (see Discussion).

### Limb constraint caused minor impairments in motor skill

**Fig 6:**
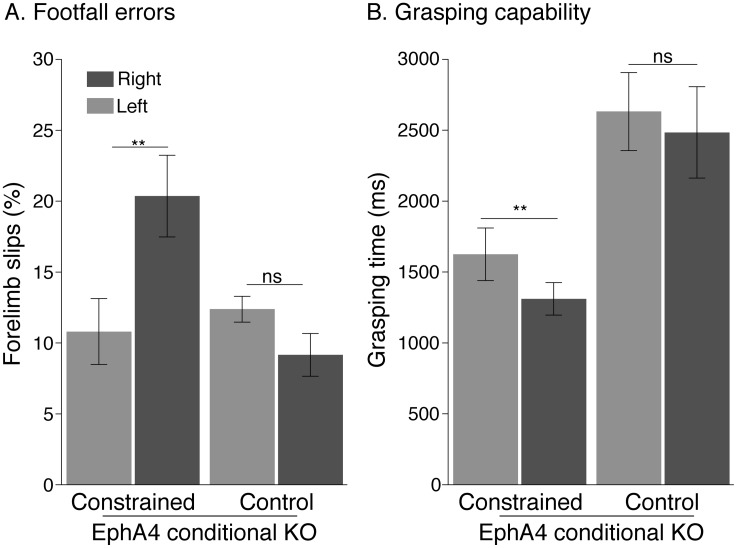
Forelimb constraint causes changes in motor skill. A. Adult mice make more errors while walking on a grid floor, with the limb bandaged during their infancy (right forelimb in this study). The bar graph shows the percent of forelimb slips was significantly higher in the constrained limb than in the unconstrained limb (*p* = 0.003, paired *t-test*). No difference was found between the two sides in the control EphA4 conditional knockout group (*p* > 0.05, paired *t-test*). Like foot placement accuracy impairment, the grasping time as shown in B was significantly shorter for the constrained than the unconstrained limb (*p* = 0.004, paired *t-test*). We found no inter-limb difference in the control EphA4 conditional knockout mice (*p* = 0.78, paired *t-test*).

We suspected that the loss of contralateral CST projections form the constrained MCX might impair motor skill. In rodents, grid walking has been shown to be a sensitive assay for limb MCX and corticospinal system function [25, 26]. We analyzed the capability to place the forelimbs accurately during spontaneous walking on a grid by measuring the percent of forelimb slips (Fig 6A). It is important to note that for this task, EphA4 conditional knockout mice do not differ from WT mice in error rate.

The error rate of the constrained limb was significantly higher than for the unconstrained limb (*p* = 0.003, paired *t-test*). We found no difference between the two sides in the EphA4 conditional knockout control group (*p* > 0.05, paired *t-test*). We also assessed maintenance of gripping/grasping capability (Fig 6B), which may assay distal limb strength. Like forepaw placement accuracy, we noted an impairment on the constrained side. Grasping time was significantly shorter for the constrained than the unconstrained limb (*p* = 0.004, paired *t-test*). Although we found no inter-limb difference in EphA4 conditional knockout control mice (*p* = 0.78, paired *t-test*), we did observe that these animals showed significantly longer grip times compared with wild type mice (2553±207.4 ms *versus* 1341±141.7 ms; *P*<0.0001, *t-test*). Our finding show a small, albeit significant, loss of motor skills for the constrained limb. The presence of a bilateral CST may confer improved strength control or relief from fatigue.

### Effect of unilateral limb constraint in wild type mice

**Fig 7:**
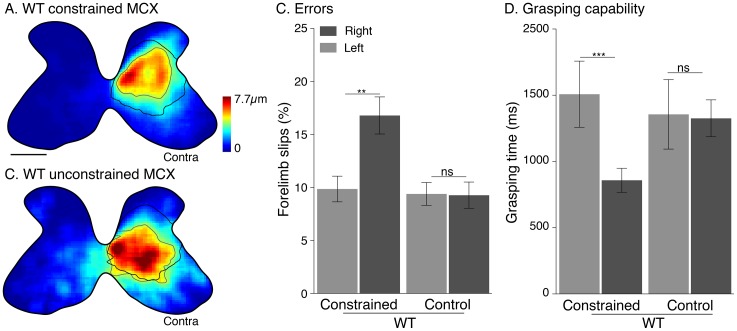
Effect of unilateral forelimb constraint in wild type mice. A, B, Heat maps for WT mice (average of 5 mice) show a significant decrease in density of CST labeling from constrained MCX (A), one-way ANOVA, *p*<0.0009, F_2, 49_ = 8.20; Bonferroni post-test: *p*<0.05). Although the CST projections from unconstrained MCX (B) show a slight expansion, there were no significant changes relative to WT controls (Bonferroni post-hoc: *p*>0.05). Lines mark contours at the yellow-green boundary. Black line is WT control contour (from Fig 2A) for comparison with the wild type constrained/unconstrained MCX (gray lines). Constrained WT show both a forelimb placement (C) and grasp capability (D) impairments (p = 0.0078, paired *t-test*, p<0.0001 paired t-test, respectively). Calibration (A) for heat maps: 250 μm.

Similar to the EphA4 conditional knockout mice, we observed a reduction in CST projections from the constrained MCX (Fig 7A). Contours mark the distribution of densest CST label in the wild type constrained (gray line) and wild type control mice (black line; from Fig 2A). There was a sparse ipsilateral projection in control wild type mice (outside contour) and even sparser in the limb constrained group. There was a 49% reduction in contralateral label and a 64% reduction in ipsilateral label (contralateral: 1846±440 pixels/axon in control group versus 947±145 pixels/axon in constrained group; ipsilateral: 264±55 pixels/axon versus 95±26 pixels/axon; one-way ANOVA; Bonferroni post-test: p<0.05).

Whereas the CST projection from the unconstrained MCX (Fig 7B) was expanded compared with the constrained MCX in wild type mice, this difference was not significant (Bonferroni posttest; *p*>0.05). The overall distributions also were similar, although we did notice that the sparse fringe of CST labeling had expanded. The lack of a substantial response from the CST from the unconstrained MCX may be due to the lack of substantial ipsilateral CST projections in wild type mice.

We also examined CST axon branching from the constrained and unconstrained MCX (see S4 Fig). Whereas we found an overall difference for the contralateral ((one-way ANOVA, *p* = 0.04, F_2,45_ = 3.4) and ipsilateral projections (one-way ANOVA, *p* = 0.005, F_2,45_ = 5.9), only the reduction in sparse ipsilateral CST projections from the constrained side showed post hoc significance (*p*<0.05, Bonferroni posttest). This suggests that, like in the EphA4 conditional knockout mice, the ipsilateral CST projections are more vulnerable than the contralateral projections. Consistent with the loss of CST projections from the constrained MCX, limb placement (p = 0.0078, paired *t-test*) and grip strength (p<0.0001 paired t-test) were both significantly impaired (Fig 7C and 7D).

As expected, unilateral limb constraint did not produce a significant difference in MCX mirror sites in wild type mice (Kruskal-Wallis test, *p* = 0.59), since there were none under control conditions. Also as expected, we found no significant difference between constrained and control wild type mice in simultaneous use of both forelimbs when reaching the wall of the cylinder, since they are so few under control conditions (*p =* 0.09, *t-test*). Importantly, and like the EphA4 conditional knockout mice, there was no significant preference for using the constrained or unconstrained limb when reaching the wall was observed in wild type controls (*p* = 0.38 paired *t-test*) or WT limb constrained mice (*p* = 0.14 paired *t-test*). The lack of any limb use preference after postnatal constraint in these mice indicates that there were no residual muscle/limb effects in maturity when we tested motor performance and assessed the motor map.

## Discussion

Using a genetic model, we showed robust and persistent motor experience-dependent development of the laterality of the CST and the function of the corticospinal system. A change in the pattern of early postnatal motor experience ameliorated the bilateral motor phenotype produced by the ipsilateral CST misprojections, brought about by conditionally deleting EphA4 in MCX [8]. Testing the animals in maturity shows that, even in the face of the conditional genetic mutation, the more lateralized CST, MCX representation, and reaching are stable. A spinal locus for the bilateral motor phenotype was demonstrated previously for this model on the basis of conditional forebrain deletion of EphA4 [8]. This resulted in a change to the developing CST and not the corticorubral projections, where we reported that contralateral projections were observed that were similar to wild-type animals [8]. This is consistent with the finding that midline expression of Ephrin-B3, a ligand for EphA4, is highest in the spinal cord, where it regulates midline axon growth, and much less so in the brain stem [27]. In our earlier study we examined quantitatively the laterality of rubrospinal and reticulospinal projections, which were shown to not be different in conditional knockout and wild type animals [8]. Similarly, in the present study the changes in spinal termination topography produced by the experience change can explain the more contralateral M1 motor map and more independent/lateralized reaching. Our findings have translational significance. Mirror movements and aberrant ipsilateral CST projections (i.e., bilateral CST) from the less impaired hemisphere are characteristic features of cerebral palsy [11, 28], a common developmental movement disorder that afflicts 2 out of 1000 live births, especially after unilateral perinatal stroke. In this way the conditional EphA4 knockout models circuit changes in cerebral palsy. The substantial normalization of the laterality of the corticospinal system by unilateral limb movement restriction reinforces the possible therapeutic benefit of constraint therapy for cerebral palsy [29, 30] on the theoretical grounds that constraint repairs CST miswiring. Further, our findings show that an important locus anatomical for the functional changes—M1 motor map and reaching laterality—is the cervical spinal cord. This also informs the pathophysiological mechanisms underlying cerebral palsy.

Anatomical and functional development of the corticospinal system occurs surprisingly late during the postnatal period in many different species, at a time when adaptive voluntary behaviors are beginning to be expressed [31–34]. Similarly, the CST connection that is thought to be the most critical for skilled movements—the monosynaptic connection with motoneurons—develops as monkeys acquire hand skills [35]; from about 3 months to 3 years [36, 37]. By contrast, systems thought to be engaged in more stereotypic control, including spinal interneuron and brain stem circuits, undergo substantial development prenatally [38–40] or before the CST [41, 42] and may be sufficiently well-specified by genetic mechanisms, needing minimal restructuring by motor experience.

### Spinal motor circuits are a locus for bilateral CST interactions

The capacity for unilateral behavioral manipulations to rewire bilaterally the corticospinal system in the conditional knockout mouse can be understood by interrelated use-dependent and activity-dependent competition for synaptic space in the spinal cord. The loss of connections can be explained by a reduction in activity-dependent synaptic competitiveness [18, 43–46]. The constrained MCX was not actively driving movements of the constrained limb between P1 and P30, and there would also be a substantial loss of phasic somatic sensory input by the reduction in limb movement. Together this amounts to a substantial reduction in feed-forward and feedback activation of the constrained motor systems. CST connections were lost bilaterally from the constrained MCX—but more so ipsilaterally, because of the reduction in the laterality index—implying differential vulnerability (discussed further below). This was corroborated by showing a substantial reduction in ipsilateral axon branching. Blocking MCX activity pharmacologically during development results in substantial loss of contralateral CST spinal projections and nearly complete elimination of ipsilateral projections [47], which is similar to the effects of constraint in the present model.

The CST from the constrained MCX showed a disproportionate loss of ipsilateral projections that resulted in a significant decrease in the CST laterality index, making the tract as contralateral as in wild type mice. The finding that the WT CST distribution, when scaled to the peak contralateral CST density (Fig 2E, inset), matched the distribution of the constrained MCX strongly suggests that the ipsilateral projection is more vulnerable than the contralateral projection. This is significant for designing therapies to abrogate the effects of ipsilateral misprojections in cerebral palsy and adult stroke [48].

Unilateral constraint produced an increase in contralateral axonal branching from the unconstrained MCX, likely driven by increased unconstrained limb use. This gain of connection is similar to the response of sensory neurons to sensory deprivation during critical periods, where thalamic neurons receiving the spared sensory inputs increase intracortical axon length and branching [21]. Strong contralateral outgrowth occurred only in the conditional knockout, where there was a concomitant loss of ipsilateral projections from the constrained MCX, resulting in opening synaptic space. In maturity, there is more substantial activity-dependent ipsilateral CST outgrowth after contralateral CST loss form the opposite hemisphere after pyramidal tract lesion [49], also implying a need to open up synaptic space. However, in maturity synaptic space would be made available by axon degeneration and the present case, by a use-dependent regression during development (competition).

We were surprised that the effect on lateralizing motor behavior was selective for reaching; bilateral “hopping” during obstructed locomotion remained unchanged. This unexpectedly distinguishes development of corticospinal circuits for reaching control from that of adaptive locomotion, which also depends on corticospinal control [8, 17, 23]. Strongly lateralized spinal circuits may be recruited during reaching, where independent limb use is required. By contrast, spinal circuits regulating right-left limb coordination during locomotion (e.g., commissural interneurons; Pitx2 interneurons [50]) are inherently bilateral, and are apt to transduce more lateralized CST signals after limb disuse into bilateral responses.

### Do ipsilateral CST misprojections contribute to impaired distal limb control?

After complete unilateral CST injury in development and maturity, corticospinal control of the affected side is from the ipsilateral CST projection [51]. Importantly, when injury occurs during development the spared ipsilateral projection achieves a substantial gain in function [52, 53]. These changes in CST circuitry are associated with contralateral impairment. We have shown in animals [54, 55] and Eyre and colleagues in humans [53] that ipsilateral CST projections from the intact hemisphere outcompete spared contralateral CST projections from the damaged hemisphere. In the human, this is associated with the progressive loss of contralateral-evoked muscle responses from M1 stimulation after injury [53]. These findings, together with the general observation that large unilateral injuries are associated with strong ipsilateral motor evoked responses, weak contralateral motor responses, and serious motor impairments on the hemiparetic side have led to view that ipsilateral CST projections spared after injury are maladaptive ([53] [56]). However, this is misleading because the gain of ipsilateral projections from the spared part of cortex is enabled by the damage of the contralateral projections from the damaged cortex. The enhancement of ipsilateral and reduction in contralateral responses occur before corticospinal motor functions are expressed [57]. These reciprocal changes are not dissociable clinically or experimentally. Thus, the question of whether the reactive increase in ipsilateral CST projections is maladaptive has not been answered by prior studies.

On each side of the spinal cord gray matter in the conditional EphA4 knockout mouse there is an abundance of ipsilateral CST projections from one hemisphere and contralateral projections from the other. The ipsilateral CST projections are functional, as shown by the bilateral motor map and bilateral forelimb muscle activation [14, 56]. However, compared with wild type mice, conditional EphA4 knockout mice have normal paw control tested in the grid task and actually have improved performance in the grip maintenance task, showing that ipsilateral CST projections are not necessarily maladaptive for some aspects of distal control. The improvement in grip maintenance performance suggests that control of a single limb by both hemispheres protects the limb from central motor systems fatigue [58]. Wild type mice after limb constraint show increases in grid walking errors and shorter grasp maintenance times. This impairment occurs with the loss of contralateral CST projections, since ipsilateral projects are negligible in these animals. After limb constraint in conditional knockout mice, grip maintenance returns to that of wild type mice showing that having more ipsilateral CST projections, when contralateral projections are maintained, is adaptive. The impairment in grid walking after constraint in conditional knockout mice was not different from the impairment seen in the wild type, again pointing to the need for contralateral projections (e.g., axon branching) for distal control and not that ipsilateral projections are maladaptive. Our findings show that it is not the gain of ipsilateral but rather the loss of contralateral projections that is maladaptive for distal control after perinatal injury. In further agreement with this result, promoting ipsilateral CST projections support behavioral improvement after complete unilateral lesion in mature animals is an effective strategy for restoring motor function [20, 59, 60].

### Harnessing use-dependent CST developmental plasticity to treat movement impairments after developmental injury

We propose that mirror movements and hand control impairments in humans after unilateral perinatal stroke [11, 48, 61] depend on circuit changes driven by spinal synaptic competition mechanisms that have gone awry. Spared projections of the damaged system are less able to drive their spinal targets, as demonstrated electrophysiologically in humans [62–64]. This leads to progressive loss of spared contralateral CST projections and a reactive increase in ipsilateral projections of the undamaged side enabled by the reciprocal processes we described above. Humans, examined using TMS [52], normally have a strongly bilateral CST before 6 months of age, with maintenance of significant ipsilateral connections. The presence of early ipsilateral CST projections to spinal motor circuits could mediate reciprocal interactions with contralateral projections, similar to the conditional knockouts we studied.

Behavioral manipulations have been used in human babies to rewire sensory and motor circuits to treat developmental disorders. Amblyopia has been treated by reducing visual experience in the eye with normal acuity to benefit the eye with reduced acuity. Although a similar approach, constraint-induced movement therapy, is used to treat developmental motor disorders, it is not fully embraced because of concerns that it could adversely affect the unimpaired/less impaired side. Using behavioral reprogramming of genetic miswiring, we show that the loss of ipsilateral misprojections is balanced by increased contralateral CST branching and outgrowth. This suggests that the ipsilateral loss is permissive for a reciprocal use-dependent increase in contralateral branching from the unconstrained MCX, and associated strengthening. These reciprocal interactions speak to the potential efficacy of behavioral modulation of CST connections after injury. In our model, unilateral limb constraint leads to more lateralized CSTs, but at a small, but significant, expense of distal skill impairments due to contralateral CST loss. Even though the disused ipsilateral CST projections may be preferentially vulnerable to elimination, how do we protect the contralateral projections? Freeing up constraint for several hours each day could be protective for limb control, similar to how providing a short period of binocular experience can protect against the loss of visual acuity produced by monocular deprivation [65, 66].

## Supporting Information

S1 Fig Representative examples if anterograde transport of CST to cervical enlargement. A-C. Each image shows an unprocessed montage of a section from one animal from each group. A. EphA4 conditional knockout control (with experience). B. EphA4 conditional knockout constrained MCX projections. C. EphA4 conditional knockout unconstrained MCX projections.
(TIF)

S2 Fig Heat maps from animals in each group. A. EphA4 conditional knockout control (with experience). B. EphA4 conditional knockout constrained MCX projections. C. EphA4 conditional knockout unconstrained MCX projections. Calibration (A) for heat maps: 250 μm.
(TIF)

S3 Fig Effect of unilateral constraint on stepping over an obstacle. Effect of limb constraint on hopping over obstacles. Histograms of forelimb locomotor behavior showed data for treadmill speed 17cm/s and obstacle height 1cm. There was a significant increase in hopping between EphA4 conditional knockout mice (n = 13–15) and WT (n = 10; one-way ANOVA, *p*<0.0001, F_2, 35_ = 15.9; Bonferroni post-hoc: *p*<0.05). However, there were no differences between the two conditional knockout groups (with experience (Ctrl.) and without experience (Constr.). Thus, limb constraint did not decrease the incidence of forelimb hopping over obstacles.
(TIF)

S4 Fig Effect of constraint on CST axon morphology in wild-type mice. A-C. CST axon morphology in controls (A), constrained MCX (B), and unconstrained MCX (C); ipsilateral (left) and contralateral (right) CST. D. Bar graphs plot CST axon branching within ROIs (inset; average of 4–5 mice/group, 4 sections/animal). Ipsilateral branching on the constrained side was significantly less than the unconstrained side and control mice (one-way ANOVA, *p* = 0.005, F_2,45_ = 5.9, Bonferroni post-hoc: *p*<0.05). No post hoc significance was obtained in the contralateral branches. (Bonferroni post-hoc: *p*>0.05).
(TIF)

S1 File Tracers, tracer efficacy correction factors, and body weight changes in wild type and EphA4 conditional knockout animals experiencing forelimb restraint. (PDF)

S2 File Matlab script for generating heat maps. (PDF)

S3 File Excel file showing data values used for plotting. Each tab contains data for the numbered figure. For Fig 2E, raw data are provided (±SEM) as this is what is plotted in the figure. For all other figures/tabs, we present mean values ± SEM values.
(XLS)
