# Supplementary figures and images for: Motor Experience Reprograms Development of a Genetically-Altered Bilateral Corticospinal Motor Circuit
Source: PLoS One. 2016 Sep 27;11(9):e0163775. doi: 10.1371/journal.pone.0163775 (PMC5038944; doi:10.1371/journal.pone.0163775)

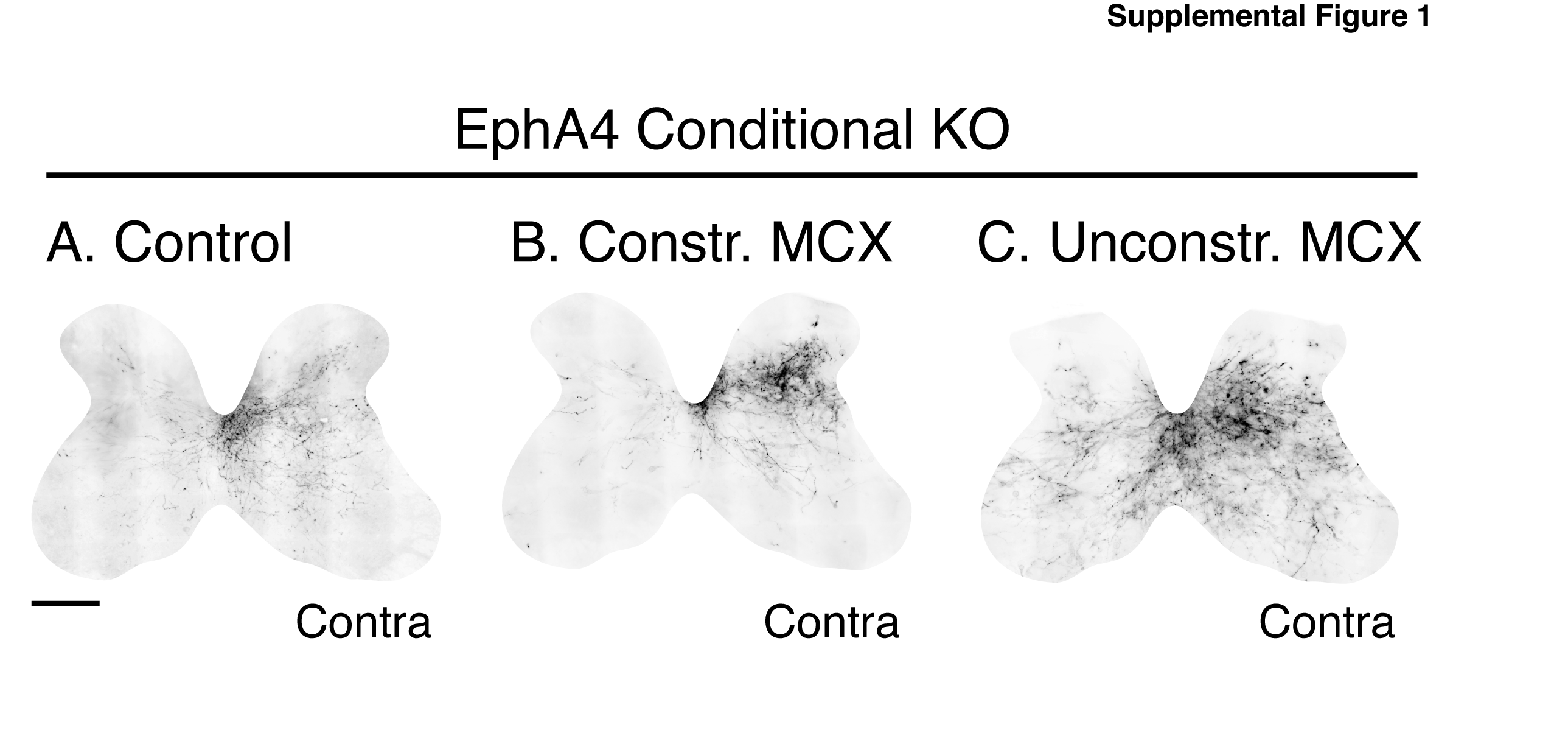

Supplement: S1 Fig — A-C. Each image shows an unprocessed montage of a section from one animal from each group. A. EphA4 conditional knockout control (with experience). B. EphA4 conditional knockout constrained MCX projections. C. EphA4 conditional knockout unconstrained MCX projections. (TIF) [file pone.0163775.s001.tif]

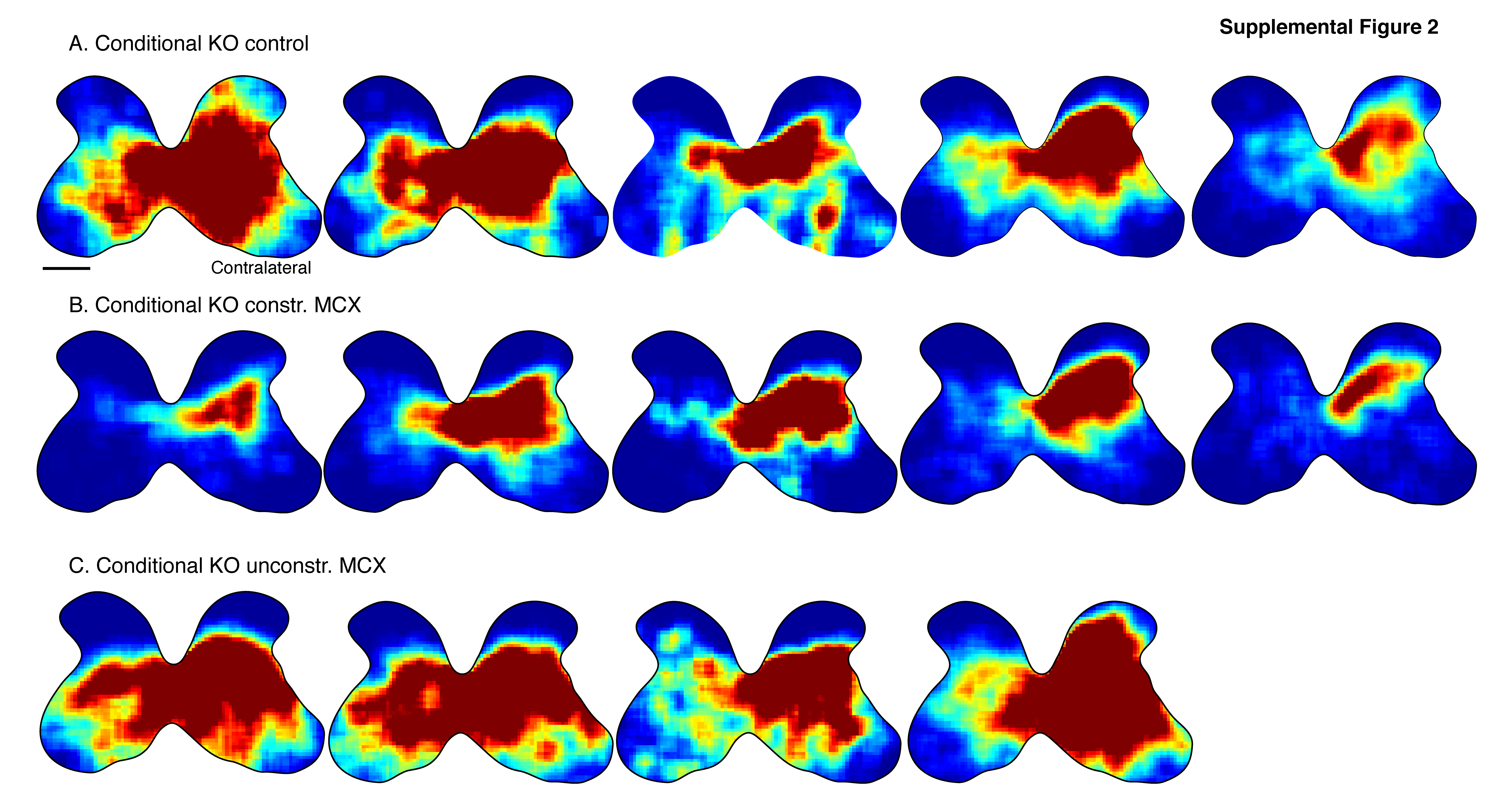

Supplement: S2 Fig — A. EphA4 conditional knockout control (with experience). B. EphA4 conditional knockout constrained MCX projections. C. EphA4 conditional knockout unconstrained MCX projections. Calibration (A) for heat maps: 250 μm. (TIF) [file pone.0163775.s002.tif]

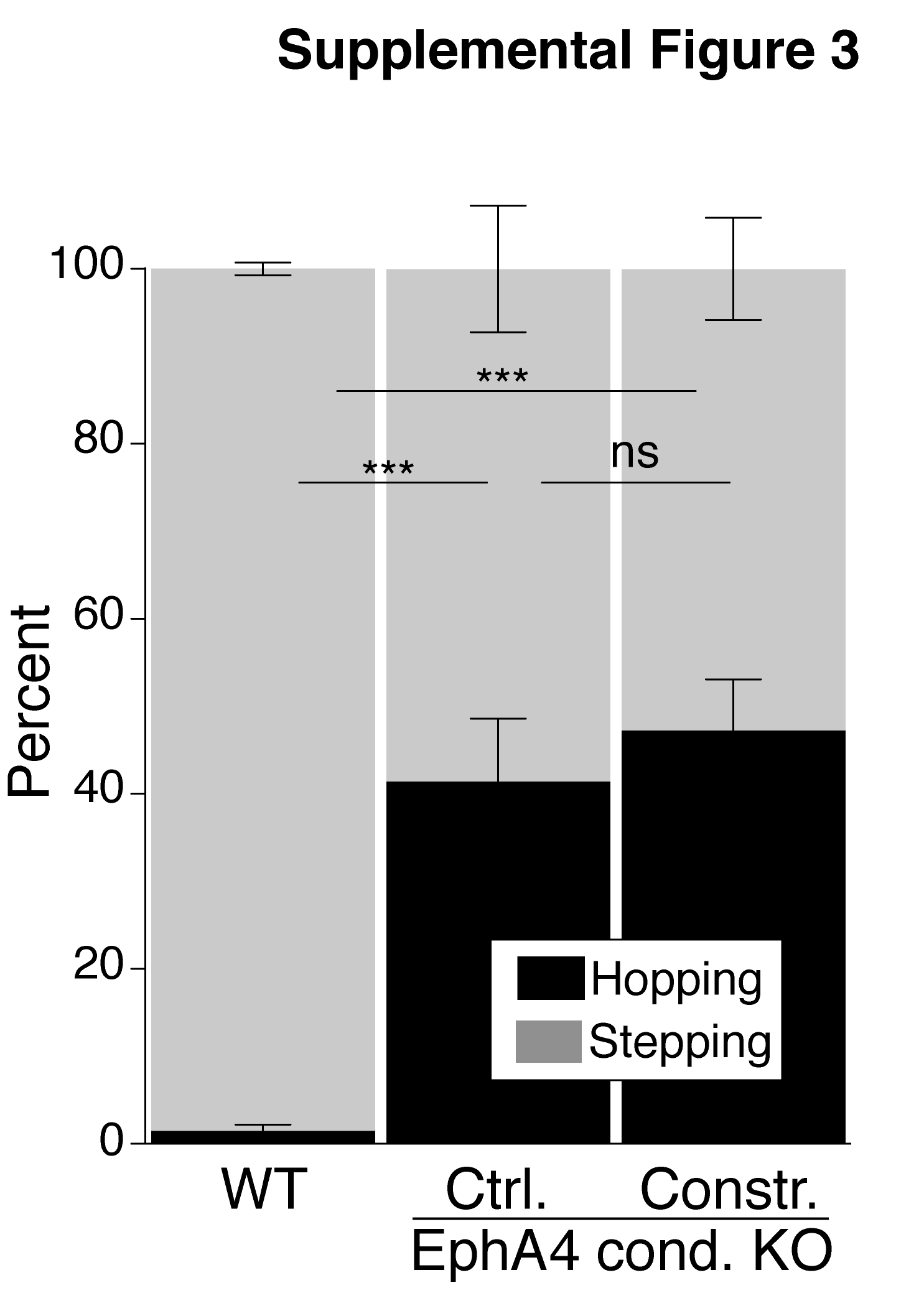

Supplement: S3 Fig — Effect of limb constraint on hopping over obstacles. Histograms of forelimb locomotor behavior showed data for treadmill speed 17cm/s and obstacle height 1cm. There was a significant increase in hopping between EphA4 conditional knockout mice (n = 13–15) and WT (n = 10; one-way ANOVA, p<0.0001, F2, 35 = 15.9; Bonferroni post-hoc: p<0.05). However, there were no differences between the two conditional knockout groups (with experience (Ctrl.) and without experience (Constr.). Thus, limb constraint did not decrease the incidence of forelimb hopping over obstacles. (TIF) [file pone.0163775.s003.tif]

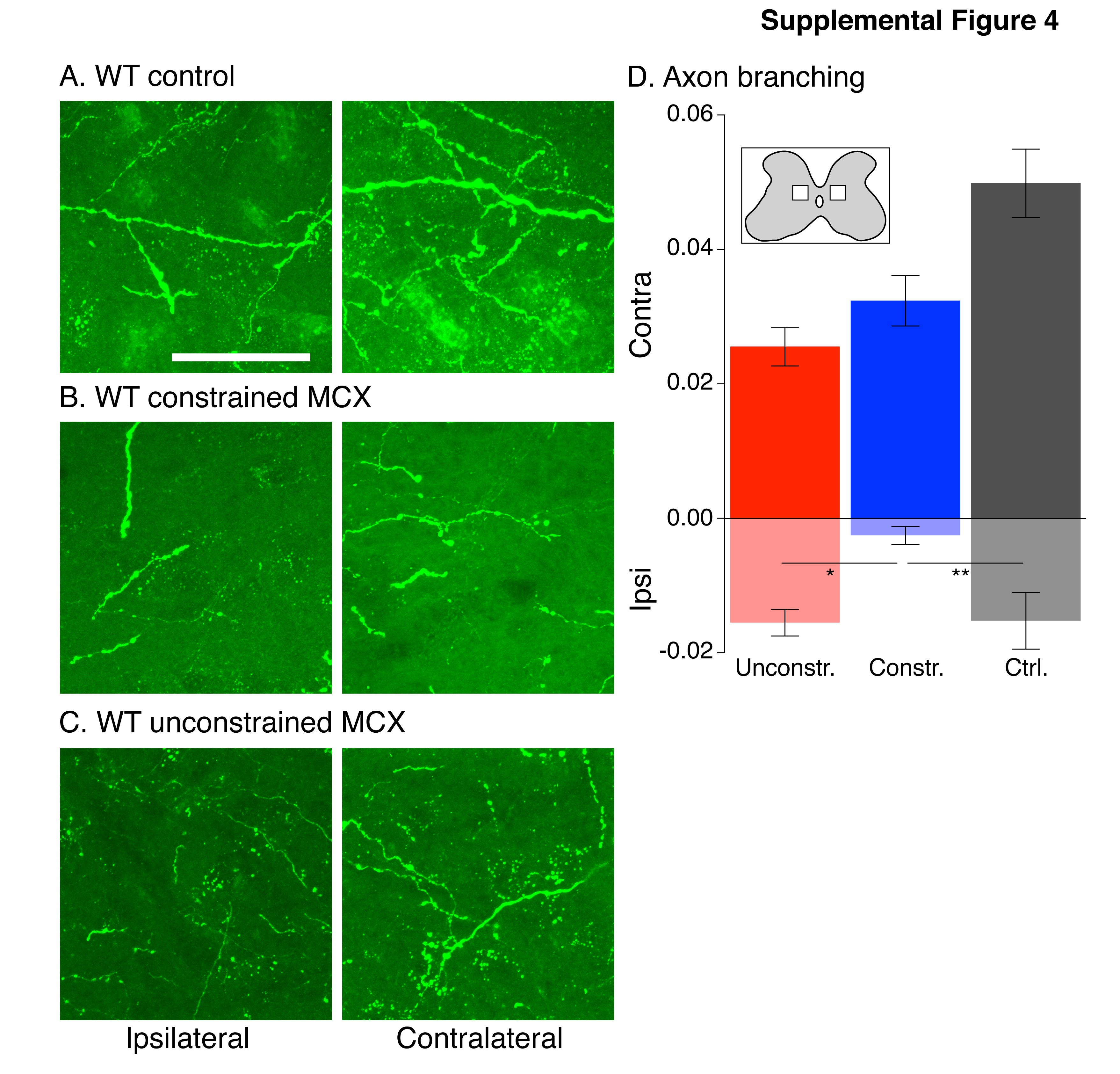

Supplement: S4 Fig — A-C. CST axon morphology in controls (A), constrained MCX (B), and unconstrained MCX (C); ipsilateral (left) and contralateral (right) CST. D. Bar graphs plot CST axon branching within ROIs (inset; average of 4–5 mice/group, 4 sections/animal). Ipsilateral branching on the constrained side was significantly less than the unconstrained side and control mice (one-way ANOVA, p = 0.005, F2,45 = 5.9, Bonferroni post-hoc: p<0.05). No post hoc significance was obtained in the contralateral branches. (Bonferroni post-hoc: p>0.05). (TIF) [file pone.0163775.s004.tif]
