# Supplementary material for: Motor Experience Reprograms Development of a Genetically-Altered Bilateral Corticospinal Motor Circuit
Source: PLoS One. 2016 Sep 27;11(9):e0163775. doi: 10.1371/journal.pone.0163775 (PMC5038944; doi:10.1371/journal.pone.0163775)

Table of tracers used for anterograde CST labeling

Group	Control/limb use	Limb constrained	
	Right M1	Right M1	Left M1
Wild type	BDA=3	BDA=3	BDA=2
	AF=2	AF=1	AF=3
Conditional KO	BDA=3	BDA=3	BDA=2
	AF=2	AF=1	AF=3

Correction factors for BDA and AF-488

We generated a "correction factor" based on the ratio of labeled CST axons in the dorsal column to the average number of labeled axons. There were no significant differences (t-test) in the correction factors/number of axons labeled for the EphA4 group (P=0.17; BDA value: 23.23± 1.986, N=31, AF488: 27.63± 2.578, N=24) or the wild type group (P=0.8; BDA value: 38.62± 1.663 N=29, AF488: 37.87± 2.598, N=23).

Body Weight

Comparison of body weights of WT disuse and EphA4 disuse groups at 4 age ranges. Importantly, there were no differences in the 7-8 week groups (t-test: P=0.38; EphA4: 17.96± 1.139, N=16; WT: 16.62 ± 0.8532, N=13). There was, however a small difference between P21 and 30 (P1-P10: P=0.025; WT: 2.93±0.09, EphA=3.27±0.1; P11-P20: P=0.8; WT: 5.24±0.09, EphA=5.28±0.15; P21-P30: P=0.026 (WT=8.21±0.2, EphA=7.5±0.24).

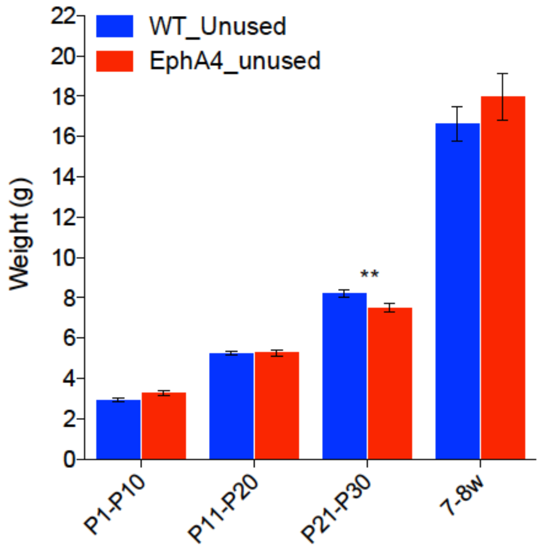

Supplement: S1 File — (PDF) [file pone.0163775.s005.pdf]
