# Supplementary material for: Motor Experience Reprograms Development of a Genetically-Altered Bilateral Corticospinal Motor Circuit
Source: PLoS One. 2016 Sep 27;11(9):e0163775. doi: 10.1371/journal.pone.0163775 (PMC5038944; doi:10.1371/journal.pone.0163775)
Supplement: S2 File — (PDF) [file pone.0163775.s006.pdf]

Matlab processing script for generating heat maps:

Listed below is the code and documentation for producing heat map topographic representations of spinal labeling.

```
function [densityImageS1, densityImageS2, densityImageSmoothS1, densityImageSmoothS2]
= Analyze(imageCellSeries1, correctionFactorSeries1, imageCellSeries2,
correctionFactorSeries2, boxSize)

% Note: the function assumes that ALL image files are of the SAME dimensions
% Parameters:
% imagesCellSeries1:  a list of images from either the same animal or from
%   the same group from different animals.
% correctionFactorSeries1: a list of integers representing the correction
%   factor.  The order of the list corresponds to the order of the images in
%   the imageCellSeries1. If a correction factor does not exist, put in the
%   value of "1" (since number/1 = number)
% For imageCellSeries2 and correctionFactorSeries2 see explanation above
% boxSize: the length, in pixels, of the SIDE of the square on which
% averaging ACROSS IMAGES will take place (vs. averaging each pixel with
% its 8 neighbors.  VERY important difference...).
%
% Syntax is as follows:
% [densityImageS1, densityImageS2, densityImageSmoothS1, densityImageSmoothS2] =
Analyze({mouse1Image1, mouse1Image2, mouse1Image3...}, {0.52, 0.69, 0.66, ...},
{mouseImage1, mouseImage2, mouseImage3,...}, {1.25, 1.53, 1.14,...}, 80}
% then, to view images, type:
% imagesc(densityImageSmoothS1)
% etc


flattenedImagesSeries1 = flattenCellsImages(imageCellSeries1, correctionFactorSeries1);
flattenedImagesSeries2 = flattenCellsImages(imageCellSeries2, correctionFactorSeries2);


% H = fspecial('gaussian',40,20);
% for currImg=1:size(flattenedImagesSeries1,2)
%     flattenedImagesSeries1{currImg} = imfilter(flattenedImagesSeries1{currImg},H,'replicate');
% end



[densityImageS1, tTestMatrixS1] = computeDensity(flattenedImagesSeries1, boxSize);
```

```
[densityImageS2, tTestMatrixS2] = computeDensity(flattenedImagesSeries2, boxSize);

% works, but excluded from script for now:
% figure, imagesc(densityImageS1);
% title('Series 1, average distribution over the entire series (correction factor applied)');
% figure, imagesc(densityImageS2);
% title('Series 2, average distribution over the entire series (correction factor applied)');
%
 aveDensImageS1 = computeNeighboringAverage(densityImageS1);
 aveDensImageS2 = computeNeighboringAverage(densityImageS2);

densityImageSmoothS1 = aveDensImageS1;
densityImageSmoothS2 = aveDensImageS2;
% figure, imagesc(aveDensImageS1);
% title('Series 1, after nearest-neighbor (per pixel) average');
% figure, imagesc(aveDensImageS2);
% title('Series 2, after nearest-neighbor (per pixel) average');
```
